# Assessment of IMU-derived lower-limb kinematics in transtibial amputees: A practical and accessible clinical workflow

**DOI:** 10.1371/journal.pone.0356786

**Published:** 2026-08-24

**Authors:** Jutima Rattanakoch, Weerawat Limroongreungrat, Gary Guerra, Udomporn Manupibul, Harit Apidech, Wisavaporn Niamsang, Mintra Suphab, Thanaphat Laucha, Manunchaya Samala

**Affiliations:** 1 Sirindhorn School of Prosthetics and Orthotics, Faculty of Medicine Siriraj Hospital, Mahidol University, Bangkok, Thailand; 2 College of Sports Science and Technology, Mahidol University, Nakhon Pathom, Thailand; 3 Department of Exercise and Sport Science, St. Mary’s University, San Antonio, Texas, United States of America; 4 School of Human Kinetics and Health, Faculty of Health Science Technology, Chulabhorn Royal Academy, Bangkok, Thailand; Polytechnic University of Marche: Universita Politecnica delle Marche, ITALY

## Abstract

Clinical gait assessment in individuals with transtibial amputation is limited by the cost, technical demands, and space requirements of laboratory-based motion analysis. Although research-grade inertial measurement unit (IMU) systems provide a portable alternative, their hardware and software costs may restrict routine clinical use. Assessment in transtibial prosthesis users is further complicated by altered segment coordination, prosthetic component function, and limited anatomical landmarks. This study evaluated a transparent, independently implemented workflow using a consumer-grade IMU-based gait analysis system (cIMU) against a research-grade reference IMU system (rIMU). Thirty-two individuals with unilateral transtibial amputation completed five overground level-walking trials while wearing both systems, with sensors mounted in standardized adjacent positions. For the cIMU workflow, segment-orientation outputs were processed using custom Python scripts to derive bilateral hip, knee, and ankle kinematics, whereas rIMU kinematics were generated using the system’s proprietary processing software. Inter-workflow agreement and repeated-trial consistency were assessed using Statistical Parametric Mapping (SPM), Bland-Altman analysis, intraclass correlation coefficients (ICCs), intra-subject variability, and paired comparisons across three anatomical planes. Repeated-trial consistency was high for hip kinematics across all three anatomical planes and for sagittal-plane knee and ankle kinematics, with average-measure ICCs ranging from 0.945 to 0.996. Inter-workflow agreement was strongest for sagittal-plane measures, with Bland-Altman mean differences within 2.5° for sagittal-plane knee and ankle measures. Wider limits of agreement were observed for several frontal- and transverse-plane measures and distal-joint variables. SPM identified significant waveform differences across multiple gait-cycle phases, particularly for frontal- and transverse-plane measures, while paired comparisons showed that differences in discrete measures were concentrated in non-sagittal and distal-joint variables. The cIMU workflow may be most useful for repeated-trial assessment of broad sagittal-plane gait patterns, whereas frontal- and transverse-plane kinematics and distal-joint measures should be interpreted cautiously. This accessible workflow may provide a practical option where research-grade gait-analysis systems are unavailable.

## Introduction

Wearable motion sensors have been increasingly used in human movement research, serving as an essential bridge between laboratory-based motion capture and routine clinical assessment of gait [1,2]. Inertial measurement units (IMUs), consisting of accelerometers, gyroscopes, and magnetometers, record inertial signals that are combined through sensor fusion algorithms to estimate segment orientation and joint kinematics in real time [3]. Their portability, relative affordability, and ability to record movement in naturalistic environments make IMUs practical tools for movement assessment outside specialized motion laboratories [4–6]. These advantages are particularly relevant to rehabilitation and prosthetic practice, where regular assessment of locomotor performance is clinically important but access to specialty motion laboratories may be limited [5,7].

Gait analysis in individuals with transtibial amputation presents methodological challenges that are not typically encountered in able-bodied individuals. Because distal bony landmarks are not fully accessible and markers or sensors are often mounted on the prosthetic socket, residual limb-socket motion and socket pistoning may affect the accuracy of kinematic measurements [7,8]. Prosthetic alignment and foot design can also alter gait mechanics and complicate the definition of segment coordinate systems [9,10]. These factors may increase mismatch between sensor-fixed and anatomical reference frames, particularly in non-sagittal planes where small alignment errors may produce kinematic cross-talk [11,12]. Previous studies have proposed strategies to improve the reproducibility of prosthesis-mounted IMU measurements, including rigid mounting plates and standardized alignment procedures [5,13]. These considerations highlight the need for measurement and processing protocols adapted specifically for IMU-based gait analysis in prosthesis users.

Although IMU technology is well established in research settings, research-grade systems remain financially inaccessible to many clinical facilities. Advances in consumer-grade IMUs have created opportunities to extend gait assessment beyond specialized laboratories [14]. However, many of these devices lack integrated biomechanical modeling or automated joint-angle computation. Consequently, external processing is required to transform sensor-orientation data into clinically interpretable joint kinematics. The technical expertise, processing time, and computational resources required for this process may limit adoption in routine clinical settings [13,15]. Developing a transparent and independently implemented processing workflow may therefore help improve the accessibility of IMU-based gait analysis while allowing the underlying computational procedures to be examined and reproduced.

Evaluating this accessible IMU-based workflow also requires an appropriate comparator. Although optical motion capture (OMC) is widely regarded as the established reference standard for biomechanical analysis, its measurement principles differ fundamentally from those of inertial sensing, and OMC measurements may be affected by soft-tissue artifact and line-of-sight limitations [16]. Previous studies have nevertheless shown acceptable agreement and reliability of research-grade IMU systems relative to OMC in individuals with transtibial amputation [5,13]. A research-grade IMU system may therefore serve as a practical benchmark for comparison within wearable sensor-based gait analysis, although it does not constitute an independent biomechanical reference standard. Despite growing interest in accessible IMU technologies, evidence remains limited regarding the agreement between consumer-grade and research-grade IMU workflows, particularly when consumer-grade sensor data are processed using a software-independent biomechanical approach.

To address this gap, the present study compared lower-limb kinematics obtained from a consumer-grade IMU-based gait-analysis workflow (cIMU) with those obtained from a research-grade IMU workflow (rIMU) in individuals with unilateral transtibial amputation. The cIMU data were processed using an established biomechanical approach that incorporated the Grood-Suntay joint coordinate system [17,18]. The study aimed to determine whether lower-limb kinematic outcomes differed between the two workflows during walking and whether agreement varied across joints and planes of motion. The null hypothesis was that no statistically significant differences would be observed between the cIMU and rIMU workflows, whereas the alternative hypothesis was that significant differences would be detected.

## Materials and methods

### Participants and ethics

Thirty-two individuals with unilateral transtibial amputation were recruited from the Sirindhorn School of Prosthetics and Orthotics, Faculty of Medicine Siriraj Hospital, Mahidol University, between September 2023 and July 2024. Sample size estimation was performed a priori using nQuery Advisor. Because the study aimed to evaluate agreement in lower-limb joint kinematics between two fixed measurement systems, the calculation was based on an intraclass correlation coefficient (ICC) framework, consistent with a two-way mixed-effects model for absolute agreement for single measurements, ICC (3,1). An ICC of 0.80 was prespecified as the minimum acceptable level of agreement, informed by previous studies reporting ICC values of 0.80 or higher for IMU-based gait kinematic measurements [19]. The required sample size was estimated for a two-sided 95% confidence interval with a prespecified precision (half-width) of 0.13, following an ICC-based reliability study design [20]. Based on these assumptions, the minimum required sample size was 31 participants. To allow for potential data loss or technical artifacts, 32 participants were recruited.

Eligible participants were adults (aged 18–75 years) with a unilateral transtibial amputation of at least six months duration and a medium-to-long residual limb. They were required to be classified as Medicare Functional Classification Levels K2–K4 and to be capable of walking independently for at least 10 meters without assistive devices. Further requirements included normal lower extremity joint range of motion, muscle strength of grade 4–5 (Oxford scale), absence of acute mobility-limiting medical conditions, and sufficient cognitive capacity to follow instructions in Thai.

Exclusion criteria included conditions that could compromise walking safety or prosthetic fit, such as residual limb complications (e.g., pain, neuromas, skin wounds, severe volume fluctuations), joint or muscle contractures, and contralateral musculoskeletal impairments. Individuals with diagnosed balance disorders or uncontrolled systemic conditions (e.g., diabetes mellitus, hypertension) were also excluded. All participants completed the trials using their routinely prescribed prostheses.

Ethical approval was granted by the Siriraj Institutional Review Board (CoA 638/2023, protocol 467/2566), and written informed consent was obtained in accordance with the Declaration of Helsinki.

### Instrumentation and sensor placement

Two IMU systems were used simultaneously during all gait trials:

1. **Consumer**-**grade IMU**-**based gait analysis system (cIMU):** Xsens DOT sensors (Movella, Enschede, Netherlands) sampling at 120 Hz. This system provides segment orientation output in quaternion or Euler-angle format without integrated biomechanical modeling [14].
2. **Research**-**grade reference IMU system (rIMU):** Noraxon MyoMotion sensors (Noraxon USA, Scottsdale, AZ) sampling at 200 Hz. The rIMU generates joint kinematics via proprietary MyoRESEARCH 3.8.1 software [5,15].

**Fig 1:**
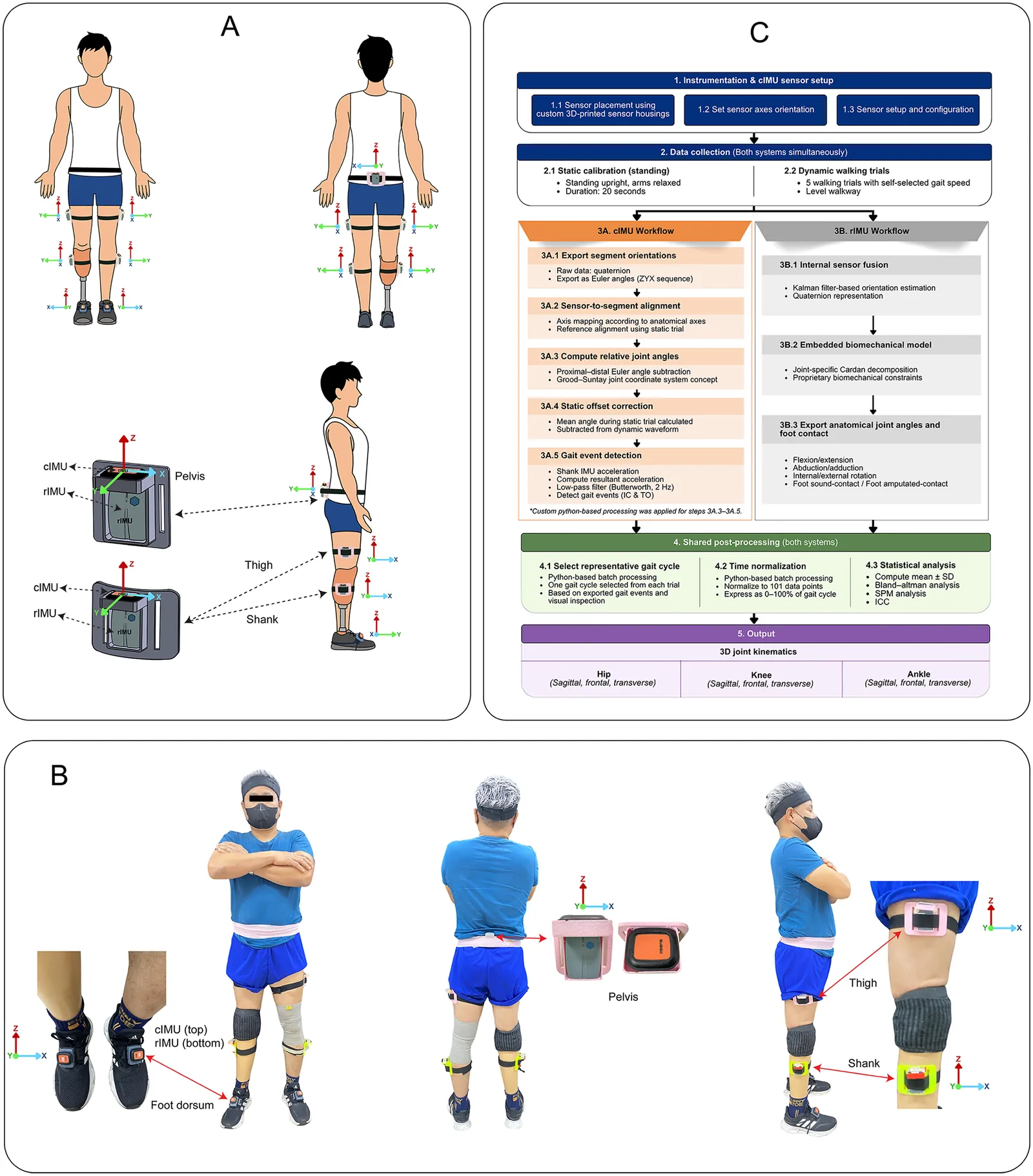
Overview of instrumentation, paired sensor placement, participant setup, and gait kinematic processing workflows. (A) Schematic views showing the adjacent placement of the cIMU and rIMU and the cIMU sensor-axis orientations. (B) Participant setup during the static standing trial. (C) Data collection and system-specific processing workflows for the cIMU and rIMU systems.

To address amputee-specific mounting constraints, sensors from both systems were positioned adjacently on the pelvis, lateral thigh, shank, and dorsum of the foot. Custom rigid 3D-printed mounting plates were used to secure the sensors and maintain consistent relative positioning at each segment. Sensor placement and mounting procedures were adapted from our previous studies comparing IMU and OMC systems [5,13]. The sensors were placed in immediate proximity to minimize differences in external sensor positioning between the two systems and to capture segment motion under closely matched conditions. In the cIMU system, mounting orientation was standardized such that the z-axis of each sensor was aligned vertically during placement to support consistent kinematic interpretation. On the dorsum of the foot, where available surface area was limited, the cIMU was mounted directly above the rIMU to preserve stable fixation and minimize positional mismatch. An overview of the sensor placement and mounting configuration is shown in Fig 1(A).

Segment coordinate systems and joint-angle descriptions were defined in accordance with established biomechanical principles for three-dimensional motion analysis and joint coordinate system interpretation [17,21]. To ensure consistency and transparency in reporting, coordinate system conventions and transformation procedures were described following current recommendations for IMU-based motion analysis [11]. For the cIMU system, segment-level orientations were obtained using the embedded Xsens Kalman Filter Core (XKFCore) sensor-fusion algorithm and exported directly as Euler (Cardan) angles using a ZYX rotation sequence (yaw-pitch-roll) relative to an earth-fixed reference frame, as specified in the manufacturer’s documentation. For the rIMU system, the exported joint kinematics were derived from segment orientations estimated using a Kalman-filter-based sensor-fusion algorithm and represented as quaternions within an embedded biomechanical model. In the present study, the 3D knee model was used. The resulting lower-limb joint angles were expressed as Cardan angles using joint-specific decomposition sequences in accordance with the International Society of Biomechanics (ISB) recommendations, where available. Specifically, hip and knee joint angles were calculated using an XZ′Y″ decomposition sequence, whereas ankle joint angles were calculated using an XY′Z″ sequence. However, the detailed implementation of the sensor-fusion procedures and biomechanical constraints remains proprietary and therefore could not be independently verified.

### Experimental protocol

Data collection was conducted along a 10-m indoor walkway. Participants wore close-fitting attire and their usual footwear. Before walking trials, a 20-second static standing trial was performed to establish baseline orientation offsets and support sensor-to-body alignment (Fig 1(B)). For the cIMU, this trial was used to define the standing reference posture and quantify these offsets for subsequent correction, whereas the rIMU followed its standard calibration procedure incorporating anthropometric measurements [14,15]. Consistent with recent work highlighting the importance of sensor-to-body calibration for anatomically meaningful IMU-based kinematics [22], this static trial served as an anatomical calibration step in the present protocol. No additional structured functional calibration movements were performed, as these may be difficult to implement consistently in individuals with transtibial amputation because of prosthetic constraints and balance limitations. Each participant completed five self-paced walking trials. Synchronization was achieved through the initial static posture and simultaneous initiation of recording on both systems.

### Computational workflow and data processing

A computational workflow was developed to derive three-dimensional gait kinematics from the cIMU. In the absence of built-in biomechanical modeling software, all kinematic parameters were derived using a software-independent processing framework. The workflow was specifically designed for gait analysis in individuals with transtibial amputation, addressing challenges related to sensor placement on prosthetic components, sensor-to-segment alignment, and reproducible computation of joint kinematics. Unlike the cIMU, the rIMU exported anatomical joint angles directly from its embedded biomechanical model. Fig 1(C) summarizes the cIMU processing workflow while indicating the point at which both systems converged to identical post-processing steps, including representative gait-cycle selection, normalization, and statistical comparison. The following subsections describe each stage of the cIMU processing pipeline in detail.

### Segment orientation and joint angle computation

Segment-level orientations were exported directly from the cIMU as Euler angles using a ZYX rotation sequence. These angles represent the mathematical decomposition of segment orientation and do not directly correspond to anatomical joint motion. Joint angles were therefore derived from the Euler angles of adjacent segments and interpreted as flexion-extension, abduction-adduction, and internal-external rotation according to the segment-specific anatomical axes defined in Table 1.

**Table 1:** Segment-specific axis definitions for joint angle interpretation in the cIMU processing pipeline.

Body Segment	Flexion/Extension (Sagittal plane)	Abduction/Adduction (Frontal plane)	Internal/External Rotation (Transverse plane)
Pelvis	X axis	Y axis	Z axis
Right/Left Thigh	Y axis	X axis	Z axis
Right/Left Shank	Y axis	X axis	Z axis
Right/Left Foot	X axis	Y axis	Z axis

For each anatomical plane, joint angles were calculated by subtracting the Euler angle of the distal segment from that of the proximal segment along the predefined axis of motion. This computation was based on the sensor-to-segment axis mapping established during sensor placement and on the assumption of consistent segment coordinate alignment throughout the trial [11,22]. The resulting joint-angle descriptions were conceptually aligned with the Grood**-**Suntay Joint Coordinate System, in which three-dimensional joint motion is interpreted with reference to anatomically meaningful axes derived from adjacent body segments [17,18,21].

Because the initial segment and joint angles were not zero during the static standing trial, a static offset correction was applied. For each variable, the mean angle obtained during the standing trial was calculated and subtracted from the corresponding dynamic waveform so that motion was expressed relative to the initial standing posture. The static recording was also used to support sensor-to-segment alignment with the gravity-defined vertical reference and to improve consistency in axis interpretation across segments [11,21,22].

**Fig 2:**
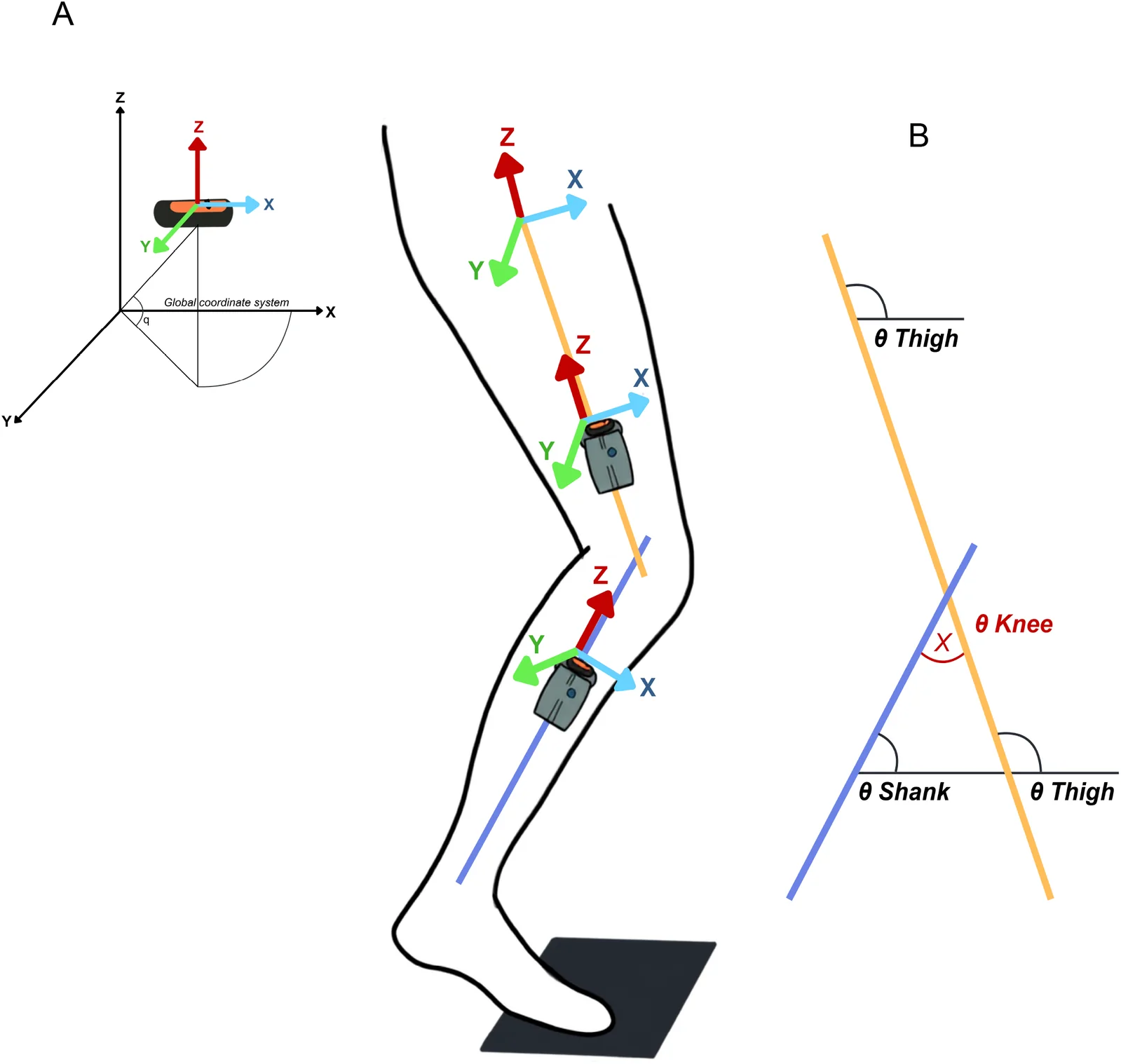
Coordinate-system definitions and knee-angle derivation in the cIMU processing workflow. (A) Relationship between the global coordinate system and the cIMU sensor axes. (B) Sensor-to-segment axis alignment and derivation of the knee flexion**-**extension angle.

For quality control, two-dimensional absolute segment angles in the sagittal and frontal planes were computed to verify axis orientation and sign conventions [21]. These planar angles served only as an internal consistency check and were not used as the primary basis for reporting joint kinematics. A conceptual illustration of the sensor-to-segment axis mapping and relative knee joint-angle computation is provided in Fig 2.

### Gait event detection and cycle selection

Gait events for the cIMU workflow were identified using resultant acceleration signals derived from the shank-mounted sensor. The resultant acceleration was computed from the tri-axial accelerometer signals as the Euclidean norm [23]:


$$\begin{array}{c} a_{resultant}=\sqrt{a_{x}^{\text{2}}+a_{y}^{\text{2}}+a_{z}^{\text{2}}} \end{array}$$

where $a_{x}$, $a_{y}$, and $a_{z}$ represent the acceleration components along the sensor’s orthogonal axes.

To suppress high-frequency noise and improve signal clarity for peak detection, the resultant acceleration signal was processed using a fourth-order Butterworth low-pass filter with a cutoff frequency of 2 Hz [24]. The sampling frequency was 120 Hz, yielding a Nyquist frequency of 60 Hz and a normalized cutoff frequency of approximately 0.033. Within each gait cycle, the filtered resultant acceleration signal consistently exhibited two prominent peaks. Initial contact (IC) was defined as the first peak, corresponding to heel-strike timing, while toe-off (TO) was identified as the subsequent peak preceding the swing phase. The transition into initial swing was further characterized by a local minimum following the TO peak [23]. Peak detection was performed using amplitude-based local maxima identification, and detected events were verified for signal quality and for a physiologically consistent temporal sequence (IC-TO-swing). In cases of irregular gait patterns or signal noise, detected events were manually corrected by selecting peaks that preserved the expected gait sequence within each cycle.

For the rIMU system, gait events were obtained from the built-in foot-contact variables exported by the MyoRESEARCH 3.8.1 software, including the “Foot Sound-Contact” and “Foot Amputated-Contact” variables. The contact-detection algorithm uses gyroscope and accelerometer data from the sensors assigned to the feet to determine the stance and swing phases using a binary on/off contact signal.

For each walking trial, one representative gait cycle was manually selected using the corresponding gait events from both systems to ensure that temporally corresponding gait cycles were compared before time normalization and waveform analysis. This yielded 160 representative gait cycles per system from 32 participants, corresponding to 320 limb-specific gait cycles per system when the amputated and sound limbs were analyzed separately. This approach maintained comparable temporal alignment between the two systems while reducing the influence of gait initiation and other irregular gait cycles.

### Normalization and data merging

Due to differing sampling rates between systems (rIMU: 200 Hz; cIMU: 120 Hz), all extracted gait cycles were time-normalized to 0–100% of the gait cycle, allowing waveform data to be compared on a common normalized time scale. This normalization was performed using linear interpolation with NumPy’s interp function. No additional filtering or smoothing was applied during the normalization and data merging step. All processing steps were performed using automated scripts written in Python 3.11 and developed in PyCharm 2024.1.4 to ensure consistent and reproducible processing of the dataset. Across both IMU systems, the final dataset comprised 640 limb-specific gait cycles. Each trial comprised 18 normalized kinematic parameters, including nine parameters for each limb and representing joint angles in three anatomical planes at the hip, knee, and ankle.

### Statistical analysis

Statistical analyses were performed to compare lower-limb kinematic measurements obtained from the cIMU and rIMU systems. Descriptive statistics were used to summarize participant characteristics, with continuous variables reported as mean ± standard deviation (SD) and categorical variables presented as frequencies and percentages.

Time-normalized joint kinematic waveforms (0–100% of the gait cycle) were analyzed using Statistical Parametric Mapping (SPM), a statistical framework for analyzing continuous biomechanical trajectories [25]. Paired-samples SPM t-tests were conducted to identify significant differences across the gait cycle. Statistical significance was set at α = 0.05, utilizing random field theory-based inference to appropriately account for multiple comparisons across the continuous time-series data.

For discrete kinematic parameters, specifically peak joint angles and range of motion (ROM) values were extracted for the hip, knee, and ankle joints across all evaluated planes. These discrete parameters were compared between the two systems using paired t-tests. To adjust for multiple comparisons across all joint-plane combinations, a Bonferroni correction was applied, setting the adjusted significance threshold at α = 0.0017.

Agreement between the systems was evaluated using Bland-Altman analysis. For each parameter, the mean difference and 95% limits of agreement (LoA) were calculated to describe the systematic differences and range of agreement between workflows [26]. Consistency between systems was further assessed using intraclass correlation coefficients based on a two-way mixed-effects model for absolute agreement, with both single-measure ICCs (ICC [1,3]) and average-measure ICCs (ICC [3, k]) reported. Single-measure ICCs reflected the reliability of an individual trial, whereas average-measure ICCs reflected the reliability of the mean across five trials. ICC values greater than 0.75 were interpreted as good, and values above 0.90 as excellent reliability [27]. Intra-subject variability was additionally calculated as the mean within-participant standard deviation across the five repeated trials [5].

All discrete statistical analyses were conducted using SPSS Statistics (version 29; IBM Corp., Armonk, NY, USA). Agreement analyses were performed using jamovi (version 2.6; The jamovi project), which operates on the R statistical computing environment (version 4.4). Waveform processing, SPM analysis, and graphical visualization were implemented using custom Python scripts (Python 3.11) within the PyCharm development environment.

## Results

### Participant characteristics

Thirty-two individuals with unilateral transtibial amputation completed the experimental protocol. Participant age ranged from 23 to 72 years (mean ± SD: 51.9 ± 13.3 years), and most were male (94%). The average body weight was 67.7 ± 13.1 kg, and the mean height was 165.7 ± 6.8 cm. Time since amputation averaged 23.2 ± 12.0 years, with a range of 3–43 years. Left-sided amputation was slightly more common (56%). Most participants had medium-length residual limbs (81%). All participants used their own endoskeletal transtibial prostheses with Patellar Tendon Bearing (PTB) sockets and either cuff suspension or suction with sleeve systems. Regarding prosthetic feet, 12 participants (37.5%) used Solid Ankle Cushion Heel (SACH) feet, 11 (34.4%) used multiaxial feet, and 9 (28.1%) used single-axis feet.

### Waveform analysis of joint kinematics using Statistical Parametric Mapping (SPM)

**Fig 3:**
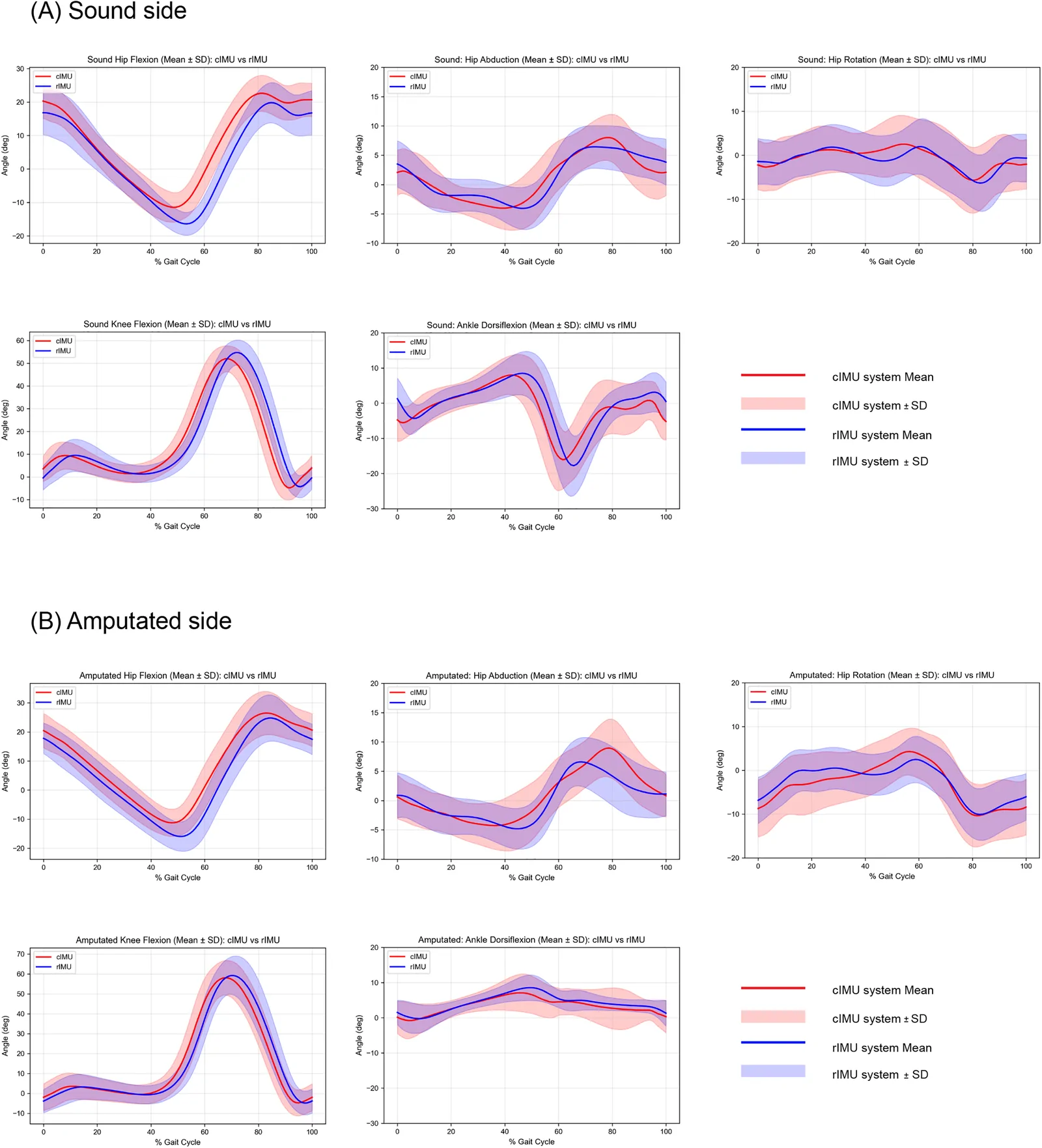
Lower-limb joint angle waveforms for the sound and amputated sides. Panels (A) and (B) show the sound-side and amputated-side results, respectively.

**Fig 4:**
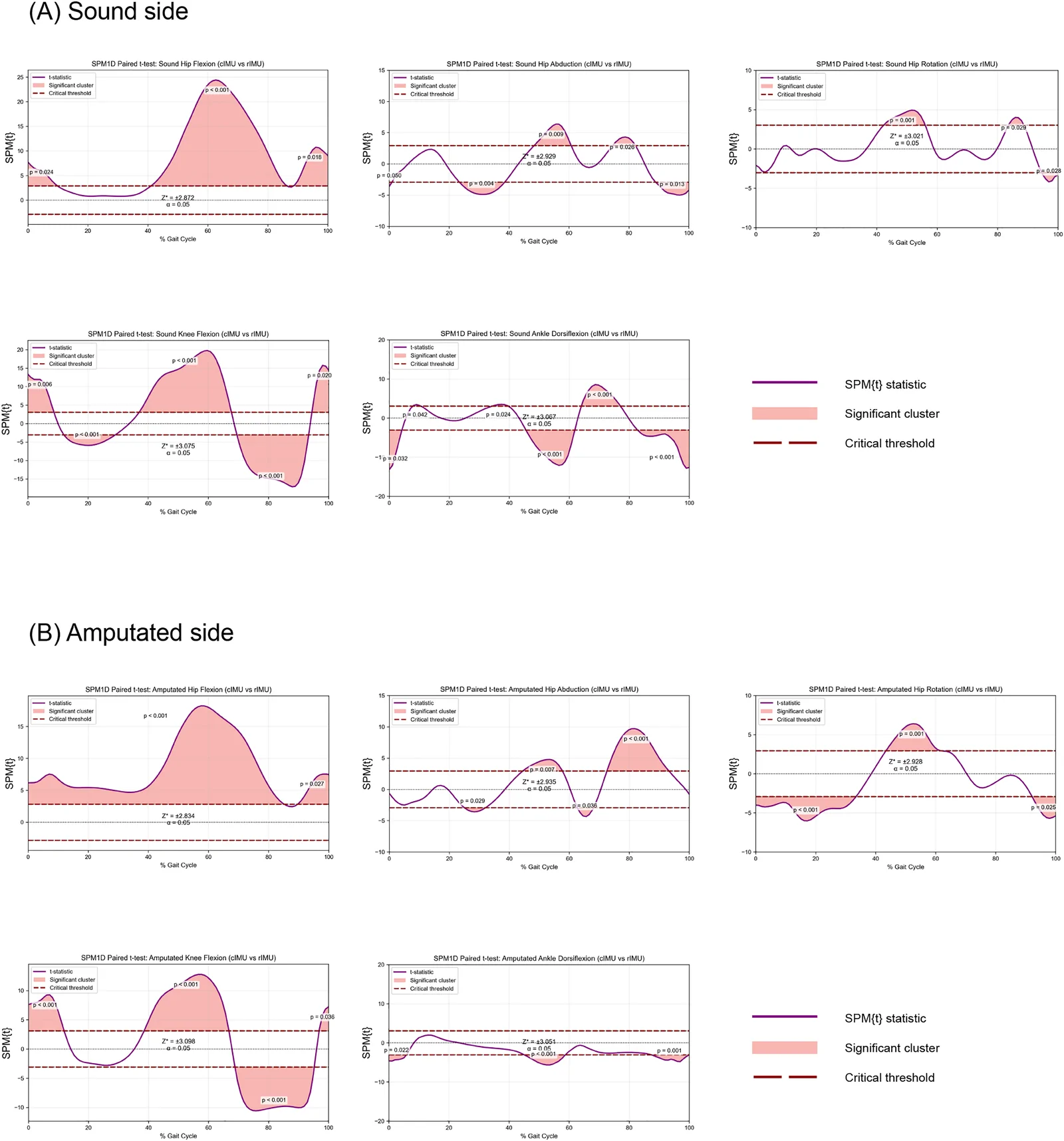
Statistical Parametric Mapping results corresponding to the joint angle waveforms inFig 3. Panels (A) and (B) show the sound-side and amputated-side results, respectively.

Joint angle trajectories for all 18 parameters, comprising nine parameters for each limb, were analyzed across the full gait cycle using Statistical Parametric Mapping (SPM). Fig 3 presents time-normalized joint angle waveforms for selected kinematic parameters from both limbs, and Fig 4 presents the corresponding SPM{t} results. These figures include hip flexion-extension, hip adduction-abduction, hip internal-external rotation, knee flexion-extension, and ankle dorsiflexion-plantarflexion. The remaining non-sagittal knee and ankle waveforms and their corresponding SPM{t} results are provided in S1 and S2 Figs, respectively.

### Sound side kinematics

At the hip joint, flexion**-**extension profiles showed significant differences during early stance (0**–**10%, p = 0.024), mid-to-terminal stance through the swing phase (41**–**85%, p < 0.001), and terminal swing (92**–**100%, p = 0.018). Hip adduction**-**abduction and internal**-**external rotation showed significant differences during mid-stance and the swing phase (p < 0.05) (Fig 4).

At the knee joint, flexion**-**extension trajectories showed significant differences during the loading response and throughout the swing phase (p < 0.001) (Fig 4). Knee adduction**-**abduction showed significant differences from 4**–**90% of the gait cycle (p < 0.001). Internal**-**external rotation showed significant differences during 5**–**32%, 62**–**80%, and 85**–**98% of the gait cycle (p < 0.01) (S2 Fig).

At the ankle joint, dorsiflexion**-**plantarflexion showed significant differences during early stance (0**–**5%), mid**-**to**-**terminal stance through the swing phase (45**–**76%), and late swing (82**–**100%; p < 0.001) (Fig 4). Inversion**-**eversion showed significant differences during multiple phases of the gait cycle, including the transition from stance to swing (p < 0.05). Ankle adduction**-**abduction showed significant differences during 4**–**28% (p < 0.001), 55**–**60% (p = 0.034), and 68**–**88% (p < 0.001) of the gait cycle (S2 Fig).

### Amputated side kinematics

At the hip joint, flexion**-**extension waveforms showed significant differences during 0**–**80% of the gait cycle (p < 0.001). Hip adduction**-**abduction and internal**-**external rotation showed significant differences during the stance-to-swing transition and mid-swing (p < 0.01) (Fig 4).

At the knee joint, flexion–extension trajectories showed significant differences during initial contact through the loading response (0**–**12%), mid-to-terminal stance (38**–**65%), and the swing phase (70**–**94% and 96**–**100%; p < 0.05) (Fig 4). Knee adduction**-**abduction showed significant differences from 6**–**90% of the gait cycle (p < 0.001), while internal**-**external rotation showed significant differences during early stance and terminal swing (p < 0.05) (S2 Fig).

At the ankle joint, dorsiflexion**-**plantarflexion trajectories showed significant differences at initial contact (0**–**6%), pre-swing (45**–**58%), and terminal swing (87**–**100%; p < 0.05) (Fig 4). Inversion**-**eversion showed significant differences at 0**–**5% (p = 0.045), 15**–**45% (p < 0.001), and 74**–**100% (p < 0.001) of the gait cycle. Ankle adduction**-**abduction showed significant differences from 10**–**60% of the gait cycle (p < 0.001) and during 84**–**98% (p = 0.003) (S2 Fig).

### Bland-Altman analysis of agreement between IMU workflows

Bland-Altman analysis was performed to assess agreement between the cIMU and rIMU measurement and processing workflows. Mean differences, 95% confidence intervals for the mean differences, and 95% limits of agreement (LoA) for all parameters are presented in S3 Table. For each parameter, the mean difference was calculated as the cIMU value minus the rIMU value; positive values indicate higher values for the cIMU workflow, whereas negative values indicate higher values for the rIMU workflow.

### Hip joint

**Fig 5:**
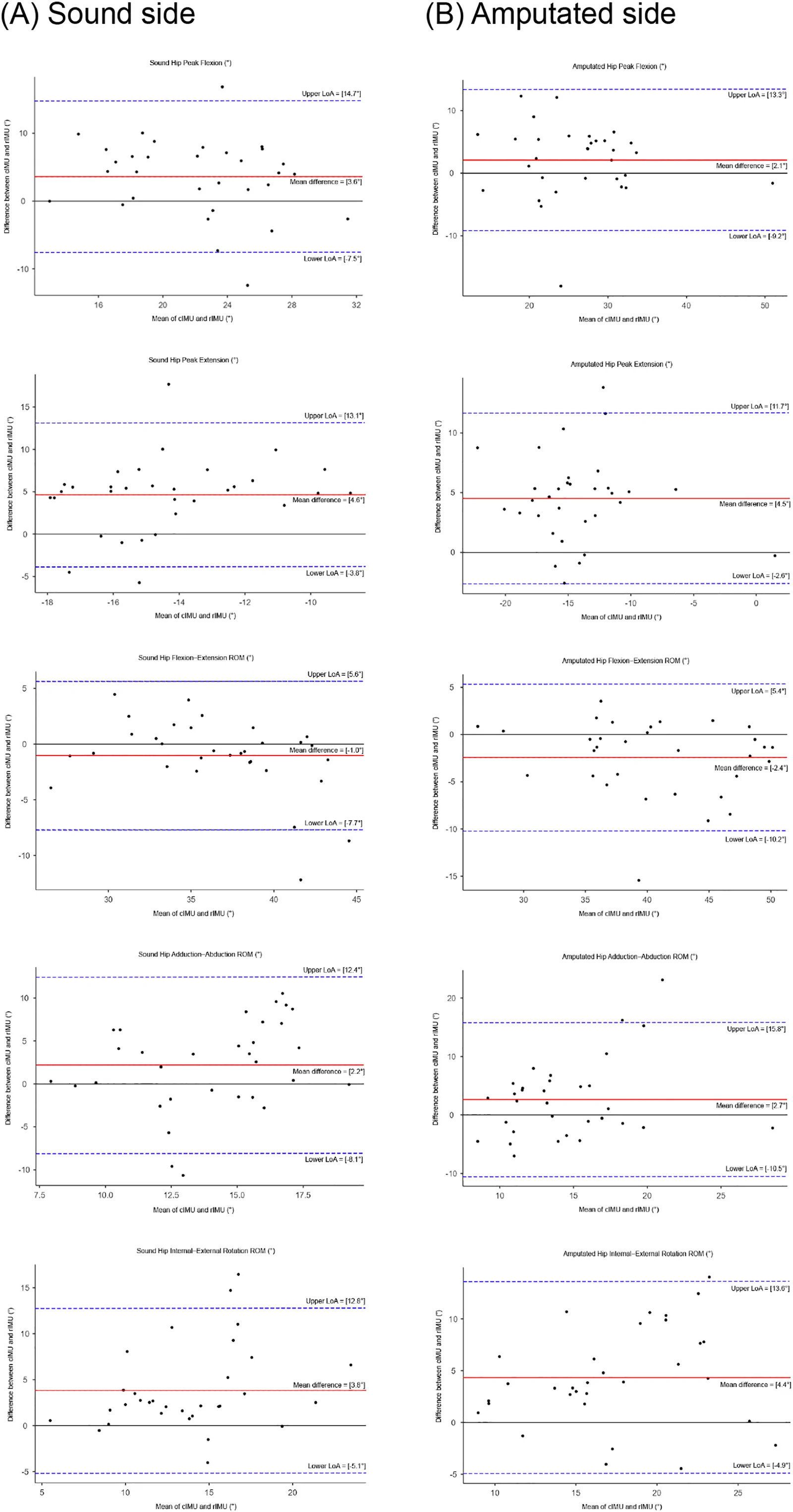
Bland-Altman plots for hip joint parameters on the sound (left column) and amputated (right column) sides. Solid red, dashed blue, and horizontal black lines indicate the mean difference, 95% limits of agreement (LoA), and zero difference, respectively.

At the hip joint, Bland**-**Altman plots for the sound and amputated sides are shown in Fig 5. On the sound side, peak hip flexion showed a mean difference of 3.6° (LoA: −7.5° to 14.7°), and peak hip extension showed a mean difference of 4.6° (LoA: −3.8° to 13.1°). Hip flexion**-**extension ROM showed a mean difference of −1.0° (LoA: −7.7° to 5.6°). Adduction**-**abduction ROM and internal**-**external rotation ROM showed mean differences of 2.2° and 3.8°, respectively. On the amputated side, peak hip flexion and peak hip extension showed mean differences of 2.1° and 4.5°, respectively. Hip flexion**-**extension ROM showed a mean difference of −2.4° (LoA: −10.2° to 5.4°). Adduction**-**abduction ROM and internal**-**external rotation ROM showed mean differences of 2.7° and 4.4°, respectively.

### Knee joint

**Fig 6:**
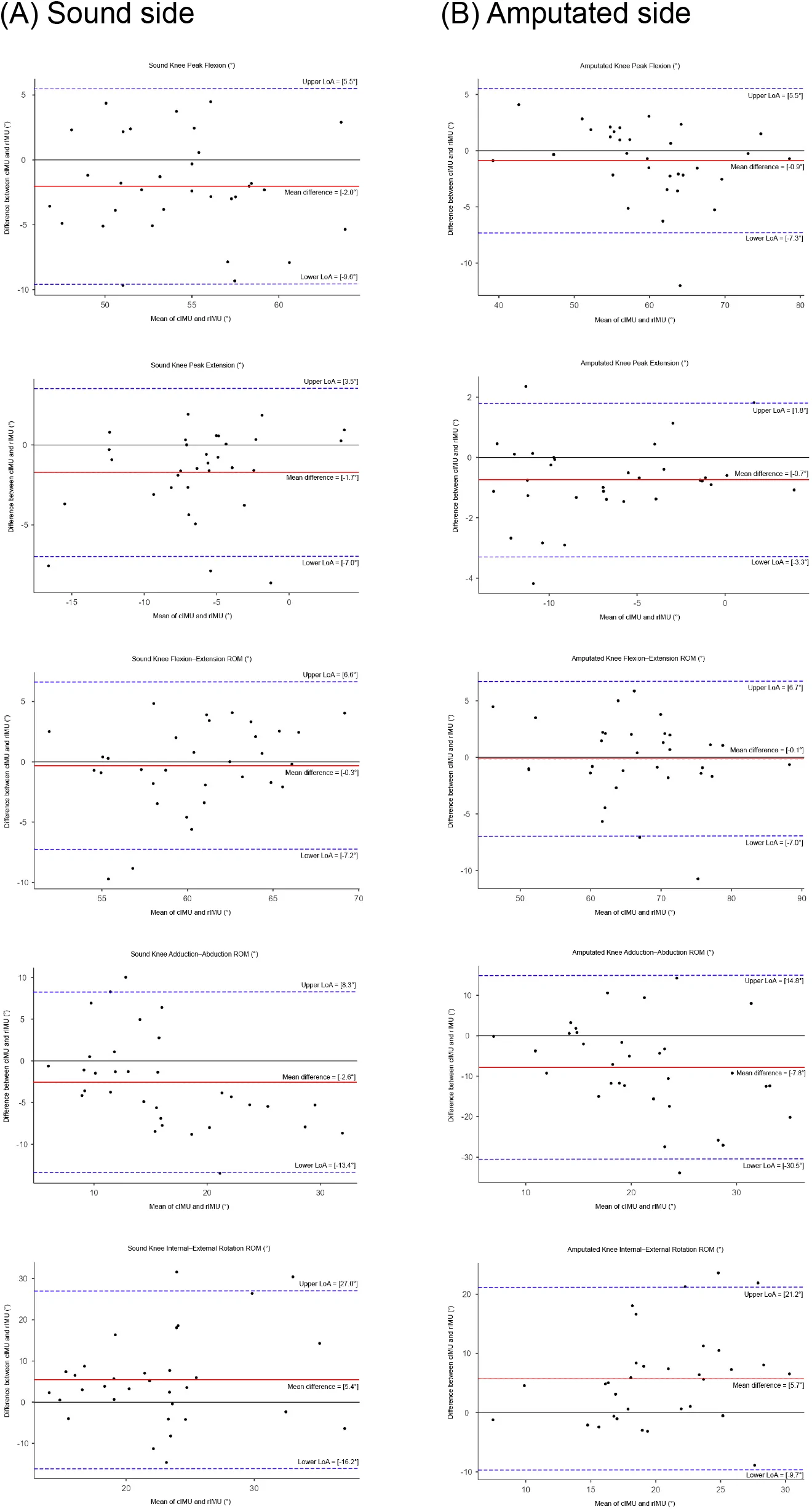
Bland-Altman plots for knee joint parameters on the sound (left column) and amputated (right column) sides. Solid red, dashed blue, and horizontal black lines indicate the mean difference, 95% limits of agreement (LoA), and zero difference, respectively.

At the knee joint, Bland**-**Altman plots for the sound and amputated sides are shown in Fig 6. On the sound side, peak knee flexion and peak knee extension showed mean differences of −2.0° (LoA: −9.6° to 5.5°) and −1.7° (LoA: −7.0° to 3.5°), respectively. Knee flexion**-**extension ROM showed a mean difference of −0.3°. Adduction**-**abduction ROM showed a mean difference of −2.6°, while internal**-**external rotation ROM showed a mean difference of 5.4° (LoA: −16.2° to 27.0°). On the amputated side, peak knee flexion and peak knee extension showed mean differences of −0.9° and −0.7°, respectively. Knee flexion**-**extension ROM showed a mean difference of −0.1°. Adduction**-**abduction ROM showed a mean difference of −7.8° (LoA: −30.5° to 14.8°), and internal**-**external rotation ROM showed a mean difference of 5.7° (LoA: −9.7° to 21.2°).

### Ankle joint

**Fig 7:**
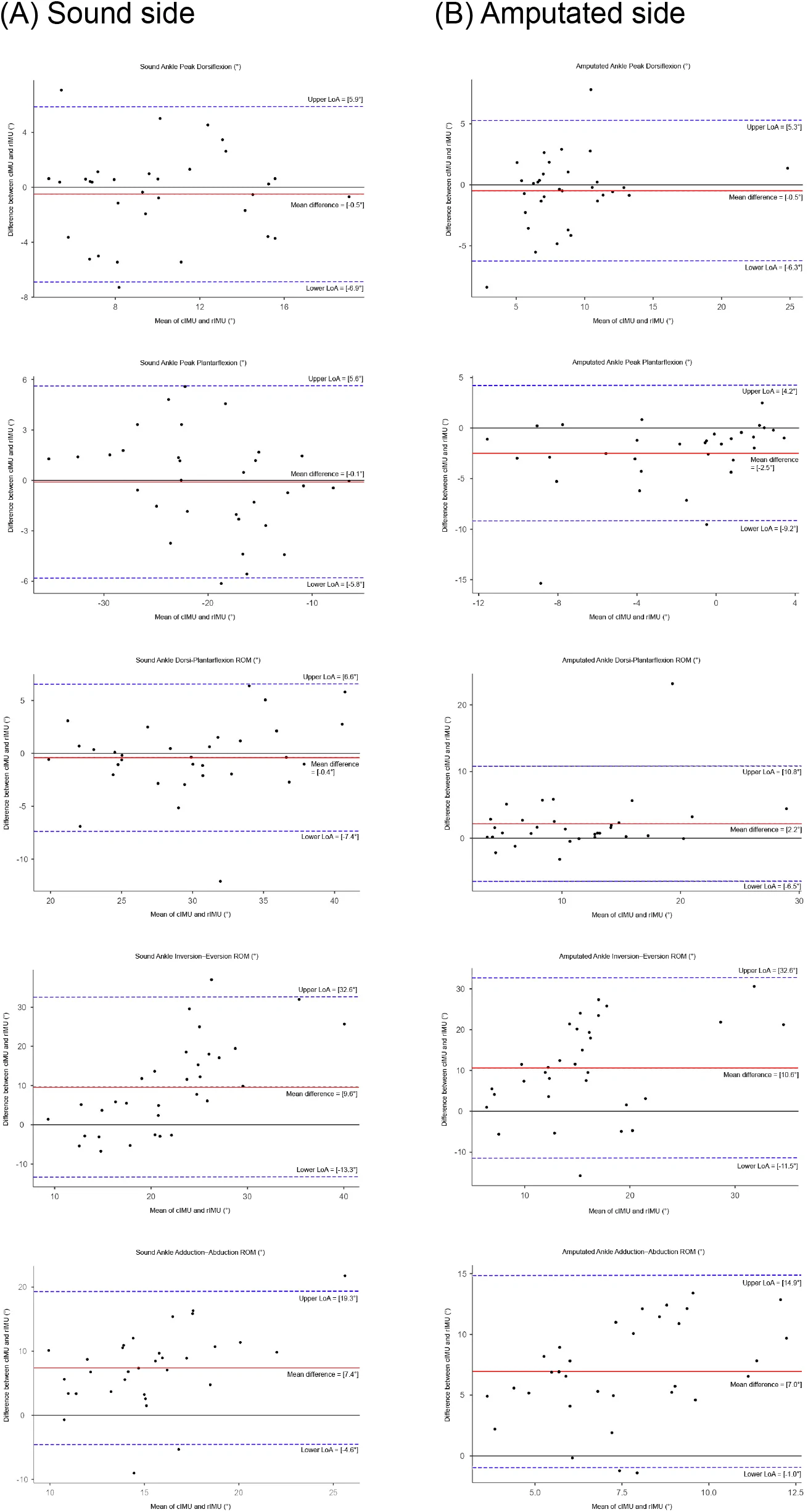
Bland-Altman plots for ankle joint parameters on the sound (left column) and amputated (right column) sides. Solid red, dashed blue, and horizontal black lines indicate the mean difference, 95% limits of agreement (LoA), and zero difference, respectively.

At the ankle joint, Bland**-**Altman plots for the sound and amputated sides are shown in Fig 7. On the sound side, peak dorsiflexion, peak plantarflexion, and dorsiflexion**-**plantarflexion ROM showed mean differences of −0.5°, −0.1°, and −0.4°, respectively. Inversion**-**eversion ROM and adduction**-**abduction ROM showed mean differences of 9.6° (LoA: −13.3° to 32.6°) and 7.4° (LoA: −4.6° to 19.3°), respectively. On the amputated side, peak dorsiflexion showed a mean difference of −0.5°. Peak plantarflexion and dorsiflexion**-**plantarflexion ROM showed mean differences of −2.5° and 2.2°, respectively. Inversion**-**eversion ROM and adduction**-**abduction ROM showed mean differences of 10.6° (LoA: −11.5° to 32.6°) and 7.0° (LoA: −1.0° to 14.9°), respectively.

### Intraclass correlation coefficient

Intraclass correlation coefficients for the comparison between the cIMU and rIMU systems are presented in Table 2. On the amputated side, single-measure ICCs ranged from 0.286 to 0.964, and average-measure ICCs ranged from 0.801 to 0.996. On the sound side, single-measure ICCs ranged from 0.273 to 0.920, and average-measure ICCs ranged from 0.790 to 0.991.

**Table 2:** Intraclass correlation coefficients for single- and average-measure lower-limb joint angles between the cIMU and rIMU workflows.

Joint Motion	Amputated Side		Sound Side	
	Single ICC	Average ICC	Single ICC	Average ICC
Hip				
Flexion/Extension	0.918	0.991	0.879	0.986
Abduction/Adduction	0.708	0.960	0.722	0.963
Internal/External Rotation	0.686	0.956	0.618	0.942
Knee				
Flexion/Extension	0.964	0.996	0.920	0.991
Abduction/Adduction	0.286	0.801	0.277	0.793
Internal/External Rotation	0.504	0.910	0.273	0.790
Ankle				
Dorsiflexion/Plantarflexion	0.634	0.945	0.775	0.972
Inversion/Eversion	0.544	0.923	0.520	0.915
Abduction/Adduction	0.328	0.830	0.280	0.796

Values are intraclass correlation coefficients (ICCs) calculated using a two-way mixed-effects model with absolute agreement to assess agreement between the cIMU and rIMU workflows. Single-measure ICCs [ICC (3,1)] indicate agreement based on individual measurements, whereas average-measure ICCs [ICC (3, k)] indicate agreement based on mean values derived from repeated measurements. ICC values were interpreted as poor (<0.50), moderate (0.50–0.75), good (0.75–0.90), or excellent (>0.90).

### Intra-subject variability

Intra-subject variability was quantified as the standard deviation across five repeated gait trials for each joint parameter within each participant, and the resulting values were averaged across participants to obtain the mean intra-subject SD (Table 3). Mean intra-subject SD values were below 4° for all evaluated parameters.

**Table 3:** Intra-subject variability of lower-limb joint kinematics across five gait trials for the cIMU and rIMU workflows.

Joint Motion Mean SD (°)	Amputated side		Sound side	
	cIMU	rIMU	cIMU	rIMU
Hip				
Flexion/Extension	1.8	1.7	2.0	1.7
Abduction/Adduction	1.6	1.2	1.5	1.2
Internal/External Rotation.	2.6	2.1	2.8	2.2
Knee				
Flexion/Extension	2.0	2.3	2.2	2.2
Abduction/Adduction	1.4	1.4	1.5	1.1
Internal/External Rotation	3.1	2.5	3.6	2.4
Ankle				
Dorsiflexion/Plantarflexion	1.4	0.7	2.7	1.8
Inversion/Eversion	1.4	1.0	2.3	2.0
Abduction/Adduction	1.1	1.2	2.2	2.7

Values represent the mean intra-subject standard deviation (Mean SD, °) of joint angles calculated across five walking trials for each participant and then averaged across participants. Higher values indicate greater trial-to-trial variability within the same system and limb side.

On the amputated side, mean intra-subject SD values ranged from 1.1° to 3.0° for the cIMU and from 0.7° to 2.5° for the rIMU. The largest numerical differences between systems were observed for knee internal-external rotation (3.1° vs. 2.5°), ankle dorsiflexion-plantarflexion (1.4° vs. 0.7°), and ankle inversion-eversion (1.4° vs. 1.0°). Ankle abduction-adduction showed a higher mean intra-subject SD in the rIMU than in the cIMU (1.2° vs. 1.1°).

On the sound side, mean intra-subject SD values ranged from 1.5° to 3.6° for the cIMU and from 1.1° to 2.4° for the rIMU. The largest numerical differences between systems were observed for knee internal-external rotation (3.6° vs. 2.4°), ankle dorsiflexion-plantarflexion (2.7° vs. 1.8°), and hip internal-external rotation (2.8° vs. 2.2°). Knee flexion-extension showed the same mean intra-subject SD in both systems (2.2°). Detailed values for all joint parameters are presented in Table 3.

### Comparison of discrete kinematic parameters using paired t-tests

Paired two-tailed t-tests were conducted to compare discrete joint-angle parameters between the cIMU and rIMU workflows. To control for Type I error due to multiple comparisons, a Bonferroni correction was applied, with the significance threshold set at p < 0.0017 (0.05/30 parameters). Statistically significant differences based on the adjusted threshold are summarized in Table 4. The complete set of comparisons, including non-significant findings, is provided in S4 Table.

**Table 4:** Statistically significant differences in discrete lower-limb kinematic parameters between the cIMU and rIMU workflows.

Joint / Side	Parameter	cIMU	rIMU	p-value
		(Mean ± SD, °)	(Mean ± SD, °)	
Hip				
Amputated	Peak stance extension	−12.0 ± 4.4	−16.5 ± 4.8	< 0.001
	Internal/External Rotation ROM	21.2 ± 6.3	17.1 ± 5.8	< 0.001
Sound	Peak stance extension	−14.3 ± 3.8	−18.6 ± 3.0	< 0.001
	Internal/External Rotation ROM	24.0 ± 7.1	18.5 ± 4.7	< 0.001
Knee				
Amputated	Abduction/Adduction ROM	18.3 ± 8.0	29.5 ± 11.2	< 0.001
Sound	Peak stance extension	−9.3 ± 5.2	−7.5 ± 4.9	0.001
	Internal/External Rotation ROM	37.3 ± 11.5	28.0 ± 7.2	< 0.001
Ankle				
Amputated	Peak swing plantarflexion	−3.7 ± 4.9	−1.5 ± 3.8	< 0.001
	Inversion/Eversion ROM	21.3 ± 10.4	13.7 ± 6.8	< 0.001
	Abduction/Adduction ROM	11.2 ± 3.8	4.4 ± 2.3	< 0.001
Sound	Peak swing plantarflexion	−25.0 ± 6.9	−22.9 ± 7.7	0.001
	Dorsiflexion/Plantarflexion ROM	38.9 ± 7.3	35.8 ± 6.0	0.001
	Inversion/Eversion ROM	35.4 ± 12.5	22.8 ± 5.2	< 0.001
	Abduction/Adduction ROM	26.2 ± 7.1	20.7 ± 7.0	< 0.001

Values are presented as mean ± standard deviation (SD) in degrees. Only parameters showing statistically significant differences after Bonferroni correction are reported in this table. The adjusted significance threshold was p < 0.0017. All other comparisons are provided in S4 Table.

At the hip, on the amputated side, the peak stance-extension angle was −12.0° for the cIMU and −16.5° for the rIMU (*p* < 0.001), indicating greater stance-phase extension in the rIMU. Internal-external rotation ROM was 21.2° for the cIMU and 17.1° for the rIMU (*p* < 0.001), indicating greater ROM in the cIMU. On the sound side, the peak stance-extension angle was −14.3° for the cIMU and −18.6° for the rIMU (*p* < 0.001), indicating greater stance-phase extension in the rIMU. Internal-external rotation ROM was 24.0° for the cIMU and 18.5° for the rIMU (*p* < 0.001), indicating greater ROM in the cIMU.

At the knee, abduction-adduction ROM on the amputated side was 18.3° for the cIMU and 29.5° for the rIMU (*p* < 0.001), indicating greater ROM in the rIMU. On the sound side, the peak stance-extension angle was −9.3° for the cIMU and −7.5° for the rIMU (*p* = 0.001), indicating greater stance-phase extension in the cIMU. Internal-external rotation ROM was 37.3° for the cIMU and 28.0° for the rIMU (*p* < 0.001), indicating greater ROM in the cIMU.

At the ankle, on the amputated side, the peak swing plantarflexion angle was −3.7° for the cIMU and −1.5° for the rIMU (p < 0.001), indicating greater plantarflexion in the cIMU. Inversion-eversion ROM was 21.3° for the cIMU and 13.7° for the rIMU (p < 0.001), while abduction-adduction ROM was 11.2° for the cIMU and 4.4° for the rIMU (p < 0.001); both ROM parameters were greater in the cIMU. On the sound side, the peak swing plantarflexion angle was −25.0° for the cIMU and −22.9° for the rIMU (p = 0.001), indicating greater plantarflexion in the cIMU. Dorsiflexion-plantarflexion ROM was 38.9° for the cIMU and 35.8° for the rIMU (p = 0.001), inversion-eversion ROM was 35.4° for the cIMU and 22.8° for the rIMU (p < 0.001), and abduction-adduction ROM was 26.2° for the cIMU and 20.7° for the rIMU (p < 0.001); all three ROM parameters were greater in the cIMU.

## Discussion

This study evaluated agreement between a clinically accessible consumer-grade IMU workflow and a research-grade IMU workflow for estimating lower-limb gait kinematics in individuals with unilateral transtibial amputation. Overall, agreement was parameter-dependent and plane-specific rather than uniform across all kinematic outcomes. Because the study compared two complete measurement and processing pipelines rather than isolated sensor outputs or either system against an optical reference standard, the findings should be interpreted as inter-workflow agreement under the study conditions, not as evidence of the absolute measurement accuracy of either system. The sample included routine prosthesis users with variation in age, time since amputation, and prosthetic foot type, although all participants used endoskeletal transtibial prostheses with patellar-tendon-bearing sockets. This variability is clinically relevant because gait in individuals with transtibial amputation may be influenced not only by biological adaptation but also by prosthetic design, suspension, and socket-residuum coupling [9,28].

Consistent with previous evidence that agreement varies across joints and planes [11,29], sagittal-plane variables generally showed smaller inter-workflow differences than frontal- and transverse-plane variables [30]. Mean differences were small for selected hip and knee flexion-extension parameters, including a knee flexion difference of less than 2.5°, and the average-measure ICCs were high (0.945–0.996). However, agreement was not consistent across all sagittal-plane variables. For the amputated-side hip, the cIMU and rIMU workflows produced broadly similar flexion-extension patterns, but SPM identified significant differences across much of the gait cycle, suggesting differences in angle magnitude and apparent timing. Other differences, including stance-phase hip extension and peak plantarflexion during swing, were restricted to specific phases of the gait cycle. The generally stronger sagittal-plane agreement may reflect the larger amplitude of flexion-extension movements and their closer alignment with the principal functional axes of the lower limbs, reducing their relative sensitivity to small orientation errors [11,19].

The observed differences in sagittal-plane waveform magnitude and timing may be partly explained by methodological differences between the workflows. The cIMU used a uniform ZYX Euler/Cardan sequence and external joint-kinematic processing, whereas the rIMU exported anatomical joint angles from a proprietary biomechanical model using joint-specific Cardan decomposition sequences. In the cIMU workflow, filtering was limited to the shank resultant acceleration used for gait-event detection, with no additional filtering or smoothing of the normalized kinematic waveforms. By contrast, internal signal-processing, optimization, and biomechanical constraint procedures within the proprietary MyoRESEARCH software may have influenced the magnitude and timing of the rIMU waveforms. Filtering and averaging procedures can reduce kinematic signal amplitude and alter the apparent timing of waveform features [31]. Differences between the custom cIMU gait-event detection procedure and the built-in rIMU foot-contact events may also have affected gait-cycle boundaries and waveform alignment, as gait-event detection accuracy depends on the algorithm, marker configuration, and gait pattern [32]. Because linear time normalization does not account for differences in detected gait-event timing, the apparent temporal shifts may reflect workflow-level differences rather than sensor delay.

In contrast, frontal-plane variables showed broader waveform differences and larger ROM mean differences, particularly at the knee and ankle. SPM indicated that knee abduction-adduction differences persisted across a substantial portion of the gait cycle on both limbs, extending from approximately 4% to 90% of the gait cycle. Ankle frontal-plane ROM also showed substantial mean differences and wide limits of agreement, ranging from nearly −13° to more than 32°. These findings are consistent with previous work showing that non-sagittal-plane kinematics are particularly sensitive to coordinate-system definitions, sensor-to-segment alignment, and sensor-to-body calibration procedures [11,22]. Because frontal-plane movements during walking generally have relatively small amplitudes, minor differences in axis definition, alignment, or rotation decomposition may have a proportionally larger effect on the resulting joint-angle estimates. Such methodological differences may also produce cross-talk, in which motion from the dominant sagittal plane is projected into the frontal plane [11]. However, the underlying sources of the observed discrepancies could not be isolated and should not be attributed solely to cross-talk. Previous IMU validation studies have similarly shown that measurement agreement varies according to anatomical region, movement task, population characteristics, and processing approach [7,16].

The largest inter-workflow discrepancies were observed in transverse-plane kinematics, as reflected by prolonged waveform differences on SPM and wide limits of agreement for rotational ROM variables. For example, sound-side knee rotation exhibited limits of agreement from −16.2° to 27.0°, and single-measure ICCs for non-sagittal variables were substantially lower than those for sagittal-plane measures. Transverse-plane kinematics are known to be sensitive to heading estimation, magnetic disturbance, and joint-coordinate definitions [11,33]. Because three-dimensional rotations are non-commutative, the workflow-specific rotation sequences described above may influence how movement is distributed across anatomical planes, particularly when transverse-plane motion is relatively small [11]. Magnetometer-derived heading in the cIMU orientation estimate may also have been affected by variability in the local magnetic environment, although environmental or prosthesis-related magnetic disturbance was not directly assessed [33]. Collectively, these methodological factors may explain the weaker agreement observed for transverse-plane measures, consistent with evidence that IMU validity varies according to the processing approach and selected gait parameters [29]. The transverse-plane findings should therefore be interpreted as inter-workflow discrepancies rather than as evidence of isolated sensor measurement error.

Measurement challenges on the amputated side may also have contributed to the larger inter-workflow discrepancies, particularly for multiplanar knee and ankle variables. For example, frontal-plane knee ROM showed a larger mean difference on the amputated side than on the sound side (−7.8° vs. −2.6°), with wider limits of agreement. This limb-specific pattern may partly reflect the complex mechanical interface of the prosthetic limb, where distal motion can be influenced by suspension, socket-residuum pistoning, prosthetic alignment, and prosthesis-related gait variability [9,28]. However, the present data cannot determine whether the observed differences arose from biomechanical adaptations, measurement-related factors, or both. Sensor location has been shown to influence lower-limb joint-angle estimates [34], whereas sensor-to-segment misalignment can substantially affect estimates of knee abduction and internal rotation [35]. Differences in sensor placement and alignment may therefore have contributed to the frontal- and transverse-plane knee discrepancies observed between the workflows. These findings also highlight the importance of interpreting ICCs alongside mean differences, limits of agreement, and waveform-level analyses, because good-to-excellent ICC values do not necessarily imply close agreement between workflows.

From a clinical perspective, both workflows showed relatively low intra-subject variability, and average-measure ICCs were consistently higher than single-measure ICCs, suggesting that assessments based on averaged repeated trials may be more informative than the detailed interpretation of a single walking trial. They also captured broad gait characteristics commonly reported in transtibial prosthesis users, including reduced hip extension during terminal stance, reduced knee flexion during swing, and limited or absent ankle plantarflexion at push-off [10,36]. The cIMU workflow may therefore be useful for assessing broad sagittal-plane gait patterns across repeated trials. However, the larger frontal- and transverse-plane discrepancies limit confidence in the detailed interpretation of mediolateral and rotational compensations. Subtle non-sagittal asymmetries should therefore be interpreted cautiously, particularly when clinical decisions depend on small angular differences [7,37].

## Limitations

Several limitations should be acknowledged. First, the analysis was restricted to level-ground walking in independently ambulatory individuals with unilateral transtibial amputation. The findings may therefore not generalize to more demanding mobility tasks, other amputation levels, socket designs, prosthetic configurations, or more diverse populations [38]. Second, this study compared two complete measurement and processing workflows and did not include an optical reference standard. Accordingly, the findings represent inter-workflow agreement rather than absolute measurement accuracy, and the observed differences cannot be attributed to sensing hardware alone. The respective contributions of sensing hardware and workflow-specific differences in kinematic processing, gait-event detection, and biomechanical modelling could therefore not be isolated. Third, although the paired sensors were rigidly secured using custom 3D-printed housings, the locations of the internal sensing elements within the commercial units were unavailable. A small spatial offset and residual sensor-to-segment misalignment may therefore have affected locally measured acceleration and three-dimensional joint-angle estimates [35,39]. Fourth, magnetometer-dependent heading estimation may have been influenced by unmeasured magnetic disturbances in the testing environment [33]. In addition, the study used a practical static calibration procedure, whereas combined anatomical and functional calibration may further improve sensor-to-segment alignment [22]. Finally, variability was evaluated across repeated trials within the same assessment session. Between-session reliability, responsiveness to clinical change, and performance during longitudinal monitoring were not examined. The workflow’s suitability for between-session and longitudinal clinical assessment therefore requires further evaluation.

## Conclusion

The study hypothesis was partially supported, as the strongest agreement between the cIMU and rIMU workflows was observed for selected sagittal-plane hip and knee kinematic outcomes in individuals with unilateral transtibial amputation, particularly when measurements were averaged across repeated trials. Agreement was less consistent for frontal- and transverse-plane measures and for several distal-joint variables, which showed larger inter-workflow differences. These findings suggest that the cIMU workflow may be useful for repeated-trial assessment of broad sagittal-plane gait patterns, whereas detailed interpretation of multiplanar and distal-joint kinematics requires greater caution. As this was a workflow-level comparison without an optical reference standard, the findings do not establish the isolated performance of the sensing hardware or the absolute accuracy of either system. Overall, the findings highlight the potential practical value of a transparent, independently implemented IMU-based gait-analysis workflow while also clarifying the parameters for which interpretation remains limited.

## Supporting information

S1 Fig Additional lower-limb joint angle waveforms for the sound and amputated sides. Panels (A) and (B) show the sound-side and amputated-side results, respectively.
(TIF)

S2 Fig Statistical Parametric Mapping results corresponding to the joint angle waveforms in S1 Fig. Panels (A) and (B) show the sound-side and amputated-side results, respectively. (TIF)

S3 Table Bland-Altman agreement analysis between the cIMU and rIMU workflows for lower-limb joint kinematics. (DOCX)

S4 Table Complete results of two-tailed paired t-tests comparing discrete lower-limb kinematic parameters between the cIMU and rIMU workflows. (DOCX)
