# Supplementary material for: Assessment of IMU-derived lower-limb kinematics in transtibial amputees: A practical and accessible clinical workflow
Source: PLoS One. 2026 Aug 24;21(8):e0356786. doi: 10.1371/journal.pone.0356786 (PMC13502542; doi:10.1371/journal.pone.0356786)
Supplement: Supplementary file 1 [file pone.0356786.s003.DOCX]

**S3 Table. Bland**-**Altman agreement analysis between the cIMU and rIMU workflows for lower-limb joint kinematics.**

| **Joint/Side** | **Parameter** | **Mean differences (°)** | | **95% CI of mean differences** | **Lower LoA (°)** | **Upper LoA (°)** |
| --- | --- | --- | --- | --- | --- | --- |
| Hip Joint (Sound Side) | Peak Flexion | 3.6 | 1.6 to 5.7 | | −7.5 | 14.7 |
|  | Peak Extension | 4.6 | 3.1 to 6.2 | | −3.8 | 13.1 |
|  | Flexion-Extension ROM | −1.0 | −2.3 to 0.2 | | −7.7 | 5.6 |
|  | Adduction-Abduction ROM | 2.2 | 0.3 to 4.1 | | −8.1 | 12.4 |
|  | Internal-External ROM | 3.8 | 2.2 to 5.5 | | −5.1 | 12.8 |
| Hip Joint (Amputated Side) | Peak Flexion | 2.1 | 0.0 to 4.2 | | −9.2 | 13.3 |
|  | Peak Extension | 4.5 | 3.2 to 5.8 | | −2.6 | 11.7 |
|  | Flexion-Extension ROM | −2.4 | −3.9 to −1.0 | | −10.2 | 5.4 |
|  | Adduction-Abduction ROM | 2.7 | 0.2 to 5.1 | | −10.5 | 15.8 |
|  | Internal-External ROM | 4.4 | 2.6 to 6.1 | | −4.9 | 13.6 |
| Knee Joint (Sound Side) | Peak Flexion | −2.0 | −3.4 to −0.7 | | −9.6 | 5.5 |
|  | Peak Extension | −1.7 | −2.7 to −0.8 | | −7.0 | 3.5 |
|  | Flexion-Extension ROM | −0.3 | −1.6 to 1.0 | | −7.2 | 6.6 |
|  | Adduction-Abduction ROM | −2.6 | −4.6 to −0.6 | | −13.4 | 8.3 |
|  | Internal-External ROM | 5.4 | 1.5 to 9.4 | | −16.2 | 27.0 |
| Knee Joint (Amputated Side) | Peak Flexion | −0.9 | −2.1 to 0.3 | | −7.3 | 5.5 |
|  | Peak Extension | −0.7 | −1.2 to −0.3 | | −3.3 | 1.8 |
|  | Flexion-Extension ROM | −0.1 | −1.4 to 1.1 | | −7.0 | 6.7 |
|  | Adduction-Abduction ROM | −7.8 | −12.0 to −3.7 | | −30.5 | 14.8 |
|  | Internal-External ROM | 5.7 | 2.9 to 8.6 | | −9.7 | 21.2 |
| Ankle Joint (Sound Side) | Peak Dorsiflexion | −0.5 | −1.7 to 0.7 | | −6.9 | 5.9 |
|  | Peak Plantarflexion | −0.1 | −1.2 to 1.0 | | −5.8 | 5.6 |
|  | Dorsi-Plantarflexion ROM | −0.4 | −1.7 to 0.9 | | −7.4 | 6.6 |
|  | Inversion-Eversion ROM | 9.6 | 5.4 to 13.8 | | −13.3 | 32.6 |
|  | Adduction-Abduction ROM | 7.4 | 5.2 to 9.6 | | −4.6 | 19.3 |
| Ankle Joint (Amputated Side) | Peak Dorsiflexion | −0.5 | −1.6 to 0.6 | | −6.3 | 5.3 |
|  | Peak Plantarflexion | −2.5 | −3.70 to −1.3 | | −9.2 | 4.2 |
|  | Dorsi-Plantarflexion ROM | 2.2 | 0.6 to 3.8 | | −6.5 | 10.8 |
|  | Inversion-Eversion ROM | 10.6 | 6.5 to 14.6 | | −11.5 | 32.6 |
|  | Adduction-Abduction ROM | 7.0 | 5.5 to 8.4 | | −1.0 | 14.9 |

Mean differences, 95% confidence intervals (CI) of the mean differences, and 95% limits of agreement (LoA) are presented in degrees. Mean differences was calculated as cIMU minus rIMU. A positive mean difference indicates that the cIMU produced higher numerical values than the rIMU, whereas a negative mean difference indicates lower numerical values. cIMU, consumer-grade IMU-based gait analysis system; rIMU, research-grade reference IMU system; ROM, range of motion; CI, confidence interval; LoA, limits of agreement.
