# Supplementary material for: Assessment of IMU-derived lower-limb kinematics in transtibial amputees: A practical and accessible clinical workflow
Source: PLoS One. 2026 Aug 24;21(8):e0356786. doi: 10.1371/journal.pone.0356786 (PMC13502542; doi:10.1371/journal.pone.0356786)
Supplement: Supplementary file 2 [file pone.0356786.s004.DOCX]

**S4 Table. Complete results of two-tailed paired t-tests comparing discrete lower-limb kinematic parameters between the cIMU and rIMU workflows.**

| **Joint / Side** | **Parameter** | **cIMU**  **(Mean ± SD, °)** | **rIMU**  **(Mean ± SD, °)** | **p-value** |
| --- | --- | --- | --- | --- |
| Hip (Amputated) | Peak swing flexion | 27.3 ± 7.4 | 25.2 ± 7.9 | 0.048 |
|  | Peak stance extension | −12.0 ± 4.4 | −16.5 ± 4.8 | <0.001* |
|  | Flex/Ext ROM | 39.3 ± 6.3 | 41.7 ± 7.0 | 0.002 |
|  | Ab/Ad ROM | 15.2 ± 4.9 | 13.5 ± 4.7 | 0.114 |
|  | Int/Ext rot ROM | 21.2 ± 6.3 | 17.1 ± 5.8 | <0.001* |
| Hip (Sound) | Peak swing flexion | 26.4 ± 5.2 | 22.9 ± 6.1 | 0.002 |
|  | Peak stance extension | −14.3 ± 3.8 | −18.6 ± 3.0 | <0.001* |
|  | Flex/Ext ROM | 40.7 ± 4.8 | 41.5 ± 6.1 | 0.183 |
|  | Ab/Ad ROM | 15.0 ± 4.6 | 13.0 ± 3.4 | 0.059 |
|  | Int/Ext rot ROM | 24.0 ± 7.1 | 18.5 ± 4.7 | <0.001* |
| Knee (Amputated) | Peak swing flexion | 59.4 ± 8.1 | 60.3 ± 9.1 | 0.142 |
|  | Peak stance extension | −7.0 ± 4.9 | −6.3 ± 4.6 | 0.003 |
|  | Flex/Ext ROM | 66.5 ± 8.9 | 66.6 ± 9.5 | 0.834 |
|  | Ab/Ad ROM | 18.3 ± 8.0 | 29.5 ± 11.2 | <0.001* |
|  | Int/Ext rot ROM | 24.5 ± 6.9 | 25.4 ± 5.8 | 0.528 |
| Knee (Sound) | Peak swing flexion | 56.8 ± 5.0 | 57.9 ± 4.9 | 0.133 |
|  | Peak stance extension | −9.3 ± 5.2 | −7.5 ± 4.9 | 0.001* |
|  | Flex/Ext ROM | 66.1 ± 5.1 | 65.3 ± 4.8 | 0.278 |
|  | Ab/Ad ROM | 19.8 ± 7.5 | 21.5 ± 8.5 | 0.082 |
|  | Int/Ext rot ROM | 37.3 ± 11.5 | 28.0 ± 7.2 | <0.001* |
| Ankle (Amputated) | Peak stance dorsiflexion | 8.9 ± 4.1 | 9.2 ± 3.7 | 0.493 |
|  | Peak swing plantarflexion | −3.7 ± 4.9 | −1.5 ± 3.8 | <0.001* |
|  | Plantar/Dorsi ROM | 12.6 ± 6.7 | 10.7 ± 5.2 | 0.017 |
|  | Inv/Ev ROM | 21.3 ± 10.4 | 13.7 ± 6.8 | <0.001* |
|  | Ab/Ad ROM | 11.2 ± 3.8 | 4.4 ± 2.3 | <0.001* |
| Ankle (Sound) | Peak stance dorsiflexion | 13.9 ± 4.1 | 12.8 ± 4.0 | 0.169 |
|  | Peak swing plantarflexion | −25.0 ± 6.9 | −22.9 ± 7.7 | 0.001* |
|  | Plantar/Dorsi ROM | 38.9 ± 7.3 | 35.8 ± 6.0 | 0.001* |
|  | Inv/Ev ROM | 35.4 ± 12.5 | 22.8 ± 5.2 | <0.001* |
|  | Ab/Ad ROM | 26.2 ± 7.1 | 20.7 ± 7.0 | <0.001* |

Values are presented as mean ± standard deviation (SD) in degrees. Results were obtained using two-tailed paired t-tests. Statistical significance was set at p < 0.0017 after Bonferroni correction, with significant differences indicated by asterisks. cIMU, consumer**-**grade IMU**-**based gait analysis system; rIMU, research**-**grade reference IMU system; ROM, range of motion; Flex/Ext, flexion**-**extension; Ab/Ad, abduction**-**adduction; Int/Ext rot, internal**-**external rotation; Inv/Ev, inversion**-**eversion.
